# Effects of *Bacillus subtilis* ZY1 on production performance, egg quality, serum parameters and intestinal health in laying hens

**DOI:** 10.1016/j.psj.2025.105120

**Published:** 2025-04-01

**Authors:** Rongrong Dong, Hao Liu, Huan Zhang, Fengyang Wu, Haidong Xiu, Shiwei Chen, Xinxiang Yin, Xiaohui Zhou

**Affiliations:** a School of Food and Biology, Hebei University of Science and Technology, Shijiazhuang 050000, PR China; b Department of Asset and Laboratory Management, Hebei University of Science and Technology, Shijiazhuang 050000, PR China; c College of Food Science and Technology, Hebei Agricultural University, Baoding 071000, PR China; d Hengshui River Animal Husbandry Co. Ltd., Hengshui, 053311, PR China; e Hebei Keweixian Biotechnology Co. Ltd., Hengshui, 053300, PR China

**Keywords:** ZY1, Laying hens, Egg quality, Serum parameter, Intestinal morphology

## Abstract

To evaluate the effects of *Bacillus subtilis* ZY1 supplementation, production performance, egg quality, serum parameters, and intestinal health in laying hens were investigated. A total of 240 healthy 59-week-old Hy-Line Brown laying hens were randomly assigned to four groups: a control group (Con) fed a basal diet and three treatment groups supplemented with *Bacillus subtilis* ZY1 at levels of 0.05 % (LG), 0.1 % (MG), and 0.2 % (HG). The duration of trial lasted eleven weeks, including a one-week pre-feeding phase. The results indicated that dietary supplementation with ZY1 increased the egg laying rate in the LG and HG groups (*P* < 0.05) as well as improved the qualified egg rate in the LG and MG groups (*P* < 0.05). Moreover, the LG group also demonstrated superior egg quality and enhanced antioxidant capacity and immune function by decreasing the level of MDA (42.47 %) and improving the content of T-AOC, GSH-Px, CAT, SOD, IgM and IgG (34.31 %, 23.92 %, 37.68 %, 31.64 %, 14.01 %, and 17.84 %, respectively) in serum samples (*P* < 0.05). The changes in biochemical parameters such as AST, LDH, TG (12.40 %, 13.79 %, and 32.13 % decreased, respectively) and Ca (41.35 % increased) were particularly pronounced in LG groups (*P* < 0.05), indicating that ZY1 supplementation enhanced metabolic capacity of laying hens (*P* < 0.05). Furthermore, laying hens in the treatment groups exhibited significantly increased villus height (VH) and an elevated villus height-to-crypt depth ratio (VH/CD) within their duodenal tissues (*P* < 0.05). These findings suggest that dietary supplementation with ZY1 effectively improves production performance, egg quality, serum parameters, and intestinal health in laying hens; notably, a dosage of 0.05 % ZY1 was identified as the optimal level for these improvements. This study provides valuable insights into optimizing the application of *Bacillus subtilis* ZY1 in laying hen management practices.

## Introduction

In poultry production, physiological issues such as decreased immunity, weakened antioxidant function, and intestinal dysfunction in laying hens often arise during the aging process, leading to reduced laying performance, diminished egg quality — including eggshell integrity and color — and occasionally increased mortality (Abudabos et al., 2019). To mitigate these challenges, antibiotics are frequently incorporated into poultry feed to sustain health status and optimize feed efficiency (Wang et al., 2023). However, antibiotic supplementation can result in bacterial resistance and residues of antibiotics in poultry products (Nechitailo et al., 2024). Furthermore, excessive use of antibiotics raises concerns regarding the potential transfer of antibiotic resistance genes from animal-derived products to humans, posing a significant threat to public health (Rafiq et al., 2022). Since July 2020, the legislation about prohibiting 11 antibiotics as feed additives has been came into effect within China (Wen et al., 2022), highlighting the need for the poultry industry to explore alternative solutions. Numerous studies indicate that certain probiotics i.e. *Bacillus subtilis* can enhance immune function in poultry, protect intestinal morphology from damage, maintain body health status, and bolster disease prevention capabilities (Yaqoob, Wang and Wang, 2022; Fathi et al., 2018; Zhang et al., 2017). Particular attention has been directed towards several probiotics utilized in chicken farming — such as *Lactobacillus* spp., *Enterococcus* spp., or *Bacillus* spp. (Forte et al., 2016). For instance, dietary supplementation with *Lactobacillus plantarum* 16 and *Paenibacillus polymyxa* 10 has shown to improve intestinal barrier function, enhance antioxidative capacity, boost immunity and reduce cell apoptosis with strain-specific effects in broilers (Wu et al., 2019). Similarly, the addition of Bonvital®, which contains viable cells of *Enterococcus faecium* DSM7134 either through feed or water administration has been effective in improving hen performance (Bampidis et al., 2020). Prior research suggested that incorporating *Bacillus subtilis* into laying hen diets could enhance production performance and maintain egg quality (Neijat et al., 2019; Guo et al., 2017). Among various probiotics studied, *Bacillus subtilis* has emerged as a favored feed additive due to its high survivability under acidic conditions, ability to produce digestive enzymes such as proteases, amylases, or cellulases, and significant potential for inhibiting the growth of pathogenic bacteria (Zou et al., 2021; Tajudeen et al., 2024; Su et al., 2020). Based on these general benefits, we focused on a specific strain, *Bacillus subtilis* ZY1, which exhibited unique antimicrobial properties. Notably, this strain can secrete CpxP protein, a regulator of the Cpx two-component system commonly found in gram-negative bacteria, which can inhibit the motility and adhesion of bacterial (Xu, Zhou and He, 2014; Zhou et al., 2011). In previous research, our group discovered that the *cpxP* gene, which encodes CpxP protein, could enhance the motility of *Escherichia coli* MG1655 after being knocked out. These findings suggested that CpxP protein played a crucial role in antimicrobial activity (He et al., 2020). Nevertheless, the role of CpxP protein in poultry remains unexplored. Therefore, in this study, ZY1 was used as the experimental strain to examine its impacts on production performance, egg quality, serum biochemical parameters, and intestinal morphology of Hy-Line Brown laying hens during the latter stages of laying period. This research aims to provide a more effective variety of feed additives for poultry farming.

## Materials and methods

### Experimental design and diets

This study was conducted in Hengshui, Hebei Province, from July to October 2023. The experimental protocols were approved by the Academic Committee of Hebei University of Science and Technology (Shijiazhuang, China) and were conducted in accordance with the National Research Council's Guide for the Care and Use of Laboratory Animals. The animal handing protocol permit number is 20200313. The ZY1 strain was isolated by the Protein Engineering Laboratory in Hebei University of Science and Technology. The ZY1 product, provided by Cangzhou Wangfa Biotechnology Research Co., Ltd. (Hebei, China), consists of spray-dried spores with a concentration of 5.4 × 10^8^ CFU/g. A total of 240 healthy 59-week-old Hy-Line Brown laying hens were randomly assigned to four groups, each consisting of three replicates with twenty hens per replicate. The control group (Con) was fed a standard corn-soybean meal basal diet (Table 1), formulated to meet the nutritional requirements for laying hens as recommended by Hy-Line International (2022). The three treatment groups received a basal diet supplemented with ZY1 at concentrations of 0.05 % (LG), 0.1 % (MG), and 0.2 % (HG), respectively. The experimental period lasted eleven weeks, including a one-week pre-feeding phase. All hens were housed in three-tier cages under controlled environmental conditions maintained at 25 ± 3°C with a daily lighting schedule of 16 h light followed by 8 h dark. Hens were fed four times daily (04:30, 09:30, 13:30, and 16:30 h) with free access to experimental diets; water was provided ad libitum via nipple drinkers continuously available.

**Table 1:** Ingredients and nutrition composition of basic diet (%).

Items	Content
Ingredients	
Corn (%)	61.1
Soybean meal (%)	23.28
Soybean oil (%)	0.97
Limestone (%)	8.73
Premix 1 (%)	1.94
Fowl galore (%)	3.88
Mold remover (%)	0.1
Total (%)	100
Nutrient level 2	
Metabolizable energy (MJ/kg)	2617
Crude protein (%)	14.9
Calcium (%)	3.52
Total phosphorus (%)	0.4
Lysine (%)	0.76
Methionine (%)	0.28

1 The premix provided the following per kilogram of diet: vitamin A 8,000 IU, vitamin D_3_ 3,300 IU, vitamin E 20 IU, vitamin B_1_ 2.5 mg, vitamin B_2_ 5.5 mg, vitamin B_6_ 4 mg, vitamin B_12_ 0.023 mg, vitamin K_3_ 2.5 mg, biotin 0.075 mg, folacin 0.9 mg, pantothenic acid 8 mg, nicotinamide 30 mg, Cu 8 mg, Fe 40 mg, Mn 90 mg, Zn 80 mg, I 1.2 mg, Se 0.22 mg.

2 The nutrient levels were calculated values.

### Egg production performance

The total number of eggs laid, egg weight, and the counts of unqualified eggs (including sand-shelled, soft-shelled, and broken) were recorded daily for each replicate during the trial. The number of qualified eggs was the total number of eggs produced minus the number of unqualified eggs. Average daily feed intake (ADFI) was measured weekly for each replicate, and the feed-to-egg ratio for each replicate was calculated by dividing total feed consumption by total egg weight. The production performance parameters — including egg laying rate, average egg weight, qualified egg rate, and feed-to-egg ratio — were computed as follows:

Egglayingrateofhens(%)=(totalnumberofeggslaidduringthestatisticalperiod)/(numberoflayinghens×numberofdays)×100%

Averageeggweight(g)=totaleggweightduringthestatisticalperiod(g)/totalnumberofeggslaid

Feed−to−eggratio=totalfeedintakeduringthetrial(g)/totaleggsweightlaid(g)

Qualifiedeggrate(%)=(numberofqualifiedeggs/totalnumberofeggs)×100%

### Egg quality

At the 11th weeks of the experiment, fifteen qualified eggs from each group were randomly collected to assess egg quality. The albumen height, yolk color, and Haugh unit were measured using an Egg Multi Tester (EA-01, ORKA Food Technology Ltd., Israel). Eggshell strength was evaluated with an Eggshell Strength Tester (EFR-01, ORKA Food Technology Ltd., Israel). Eggshell thickness was assessed using an Egg Shell Thickness Gauge (TI-PVX, ORKA Food Technology Ltd., Israel), with measurements taken at the blunt end, tip end, and middle of each egg averaged to calculate overall eggshell thickness. Additionally, eggshell weight, yolk weight, and albumen weight were recorded using an electronic balance. Furthermore, eggshell color values [L* (lightness), a* (redness), b* (yellowness)] were determined utilizing an Eggshell Color Tester (NH310, Shenzhen San'enshi Technology Co., Ltd., Shenzhen, Guangdong Province, China).

### Serum parameters

At the end of the trial, nine laying hens per group were randomly selected for serum parameter analysis following a 12-hour feed withdrawal. Blood samples (approximately 5 mL per hen) were collected from the wing vein using disposable needles. Serum samples were obtained by centrifuging the blood at 3000 rpm for 15 min and subsequently stored in sterilized 1.5 mL Eppendorf tubes at −20°C until analysis.

The levels of total antioxidant capacity (T-AOC), glutathione peroxidase (GSH-Px), catalase (CAT), total superoxide dismutase (T-SOD), and malondialdehyde (MDA) concentration in serum were measured using commercial kits (Nanjing Jiancheng Bioengineering Institute, Nanjing, Jiangsu, China), according to the manufacturer's instructions. Similarly, immunoglobulin A (IgA), immunoglobulin M (IgM), immunoglobulin G (IgG), aspartate aminotransferase (AST), alanine aminotransferase (ALT), lactic dehydrogenase (LDH), triglycerides (TG), glucose (GLU), calcium (Ca), and phosphorus (P) were determined utilizing commercial kits (Nanjing Jiancheng Bioengineering Institute, Nanjing, Jiangsu, China), via colorimetric methods on a semi-automatic biochemical analyzer (L-3180, Shanghai Kehua Bio-Engineering co., Ltd., Shanghai, China), according to the manufacturer's protocols.

### Intestinal morphology

At the end of the experiment, nine hens from each group were randomly selected and euthanized by cervical dislocation. Approximately 3 cm segments of the middle duodenum, jejunum, and ileum were excised and gently flushed with 0.9 % NaCl several times to remove digestive contents. Subsequently, these samples were fixed in 4 % paraformaldehyde for 24 h. Following fixation, intestinal samples underwent routine processing including dehydration and clearing before being embedded in paraffin wax. Once the wax blocks were prepared, tissue sections measuring 3 μm in thickness were cut and stained with hematoxylin and eosin prior to mounting with neutral resin. Intestinal morphology was examined using an optical microscope (DM2500, Leica Microsystems, Germany).

The villus height (VH) and crypt depth (CD) were measured using ImageJ software, allowing for calculation of the VH/CD ratio. Nine intact intestinal villi values from each group were randomly selected to compute average values for data analysis.

### Statistical analysis

All results were analyzed using one-way ANOVA with SPSS 27.0 software (SPSS Inc., Chicago, IL). Data are presented as means ± SEM in the tables and figures. *P* < 0.01, *P* < 0.05, and 0.05 < *P* < 0.10 were considered to indicate high statistical significance, significance, and a trend, respectively.

## Results

### Effects of dietary ZY1 on egg production performance

As shown in Table 2, while there was no significant change in average egg weight, dietary supplementation with ZY1 in the LG and HG groups significantly improved the egg laying rate (*P* < 0.05). Additionally, throughout the experimental period, the feed-to-egg ratio of the HG group was significantly lower than that of the Con group (*P* < 0.05). Notably, ZY1 supplementation significantly increased the qualified egg rate for both the LG and MG groups (*P* < 0.05).

**Table 2:** Effects of ZY1 on egg production performance of late laying hens.^1^.

Item	0 (Con)	0.05 % (LG)	0.1 % (MG)	0.2 % (HG)	*P*-value
Egg laying rate (%)	69.47 ± 0.47^c^	72.03 ± 1.01^b^	68.64 ± 0.44^c^	74.48 ± 0.90^a^	<0.001
Average egg weight (g)	64.59 ± 0.10	64.96 ± 0.12	64.59 ± 0.09	64.76 ± 0.14	0.085
Feed-to-egg ratio	2.63 ± 0.02^a^	2.57 ± 0.03^a^	2.60 ± 0.01^a^	2.48 ± 0.03^b^	<0.001
Qualified egg rate (%)	79.98 ± 1.07^b^	87.90 ± 1.20^a^	85.41 ± 1.05^a^	81.13 ± 1.21^b^	<0.001

^a,b^ Within a row, values with no common superscripts indicate a significant difference (*P*<0.05).

1 Data are expressed as mean ± SEM, *n* = 60.

### Effects of dietary ZY1 on egg quality

The effects of ZY1 supplementation on egg quality parameters are summarized in Table 3. Over the 11-week period, significant differences were observed in eggshell weight, yolk weight and eggshell brightness (L*) in the low and high dose dietary ZY1 groups compared to the Con group (*P* < 0.05). Additionally, all three ZY1 supplementation groups exhibited a significant increase in eggshell redness (a*) relative to the Con group (*P* < 0.01). Furthermore, a decreasing trend in eggshell yellowness (b*) was noted in the treatment groups compared to the Con group (*P* < 0.10).

**Table 3:** Effects of ZY1 on the egg quality of late laying hens.^1^.

Item	0 (Con)	0.05 % (LG)	0.1 % (MG)	0.2 % (HG)	*P*-value
Shell thickness (mm)	0.46 ± 0.01	0.46 ± 0.01	0.46 ± 0.01	0.47 ± 0.01	0.385
Shell strength (N/m^2^)	41.28 ± 2.06	39.62 ± 1.81	39.60 ± 1.33	41.23 ± 1.25	0.800
Shell weight (g)	6.35 ± 0.15^c^	6.76 ± 0.11^ab^	6.49 ± 0.09^bc^	6.88 ± 0.08^a^	0.005
Albumen weight (g)	42.01 ± 0.56	42.50 ± 1.05	42.96 ± 0.70	42.45 ± 0.78	0.870
Albumen height (mm)	5.87 ± 0.41	5.80 ± 0.49	6.61 ± 0.31	6.48 ± 0.35	0.351
Yolk weight (g)	16.79 ± 0.41^b^	17.82 ± 0.25^a^	16.40 ± 0.33^b^	17.78 ± 0.31^a^	0.005
Yolk color	6.47 ± 0.35	6.87 ± 0.39	5.93 ± 0.36	6.40 ± 0.53	0.471
Haugh unit	70.79 ± 3.99	67.82 ± 5.61	77.30 ± 2.71	76.05 ± 2.58	0.286
L*	60.49 ± 1.40^a^	55.66 ± 0.82^b^	58.16 ± 0.77^ab^	57.54 ± 0.63^b^	0.008
a*	19.39 ± 0.49^b^	20.62 ± 0.38^a^	20.73 ± 0.18^a^	21.03 ± 0.13^a^	0.005
b*	24.53 ± 0.63	25.74 ± 0.38	26.17 ± 0.27	25.42 ± 0.29	0.051

^a,b^ Within a row, values with no common superscripts indicate a significant difference (*P*<0.05).

L* = lightness; a* = redness; b* = yellowness.

1 Data are expressed as mean ± SEM, *n* = 15.

### Effects of dietary ZY1 on antioxidant capacity

**Fig. 1:**
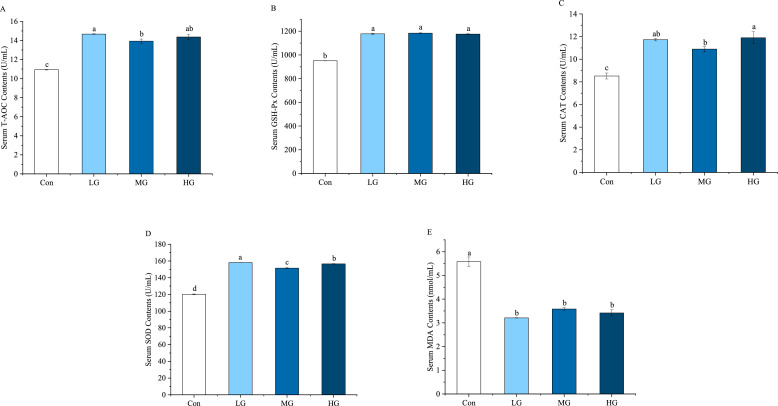
Effects of ZY1 on antioxidant indices of late laying hens. (A) The contents of T-AOC in serum, (B) The contents of GSH-Px in serum, (C) The contents of CAT in serum, (D) The contents of T-SOD in serum, (E) The contents of MDA in serum. ^a-c^ Values in a row with no common letters indicate a significant difference (*P*<0.05). Note: *n* = 9.

The effects of ZY1 on antioxidant capacity are illustrated in Fig. 1. Levels of T-AOC, CAT, GSH-Px and SOD were significantly improved among the different treatment groups compared to the Con group (*P* < 0.05). Moreover, MDA level was significantly reduced in the ZY1-treated group (*P* < 0.05), indicating enhanced antioxidant capacity.

### Effects of dietary ZY1 on immune indices

**Fig. 2:**
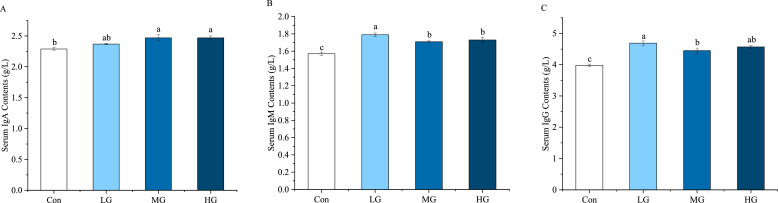
Effects of ZY1 on immune indices of late laying hens. (A) The contents of IgA in serum, (B) The contents of IgM in serum, (C) The contents of IgG in serum. ^a-c^ Values in a row with no common letters indicate a significant difference (*P*<0.05). Note: *n* = 9.

The effect of ZY1 supplementation on immune indices are presented in Fig. 2. In the MG and HG groups levels of IgA were significantly higher than those observed in the Con group (*P* < 0.05). Furthermore, serum IgM and IgG levels were significantly elevated in all three ZY1 supplementation groups relative to the Con group (*P* < 0.05).

### Effects of dietary ZY1 on biochemical parameters

The effects of dietary ZY1 on biochemical parameters are summarized in Table 4. Compared to the Con group, levels of AST and TG were significantly reduced in the LG and MG groups, while ALT levels exhibited a decrease in the HG group (*P* < 0.05). Additionally, within the treatment groups, LDH levels were lower than those observed in the Con group, whereas calcium (Ca) levels were higher compared to the Con group in serum (*P* < 0.01).

**Table 4:** Effects of ZY1 on biochemical parameters of late laying hens.^1^.

Item	0 (Con)	0.05 % (LG)	0.1 % (MG)	0.2 % (HG)	*P*-value
AST (U/L)	173.23 ± 0.90^b^	151.75 ± 0.80^d^	168.08 ± 0.38^c^	185.75 ± 0.43^a^	<0.001
ALT (U/L)	30.70 ± 0.61^c^	37.51 ± 0.35^a^	34.36 ± 0.23^b^	22.59 ± 0.23^d^	<0.001
LDH (U/L)	1150.81 ± 4.54^a^	992.10 ± 5.51^b^	954.66 ± 1.24^c^	985.10 ± 1.06^b^	<0.001
TG (mmol/L)	11.39 ± 0.33^a^	7.73 ± 0.16^b^	8.52 ± 0.28^b^	11.35 ± 0.23^a^	<0.001
Ca (mmol/L)	7.40 ± 0.50^c^	10.46 ± 0.20^a^	9.73 ± 0.17^a^	8.40 ± 0.27^b^	<0.001
GLU (mmol/L)	10.89 ± 0.17	11.25 ± 0.52	11.09 ± 0.26	11.88 ± 0.47	0.355
P (mmol/L)	1.96 ± 0.14	1.50 ± 0.19	1.46 ± 0.07	1.41 ± 0.09	0.060

^a,b^ Within a row, values with no common superscripts indicate a significant difference (*P*<0.05).

Abbreviations: AST, aspartate aminotransferase; ALT, alanine aminotransferase; LDH, lactic dehydrogenase; TG, triglycerides; GLU, glucose; Ca, calcium; P, phosphorus.

1 Data are expressed as mean ± SEM, *n* = 9.

### Effects of dietary ZY1 on intestinal morphology

**Fig. 3:**
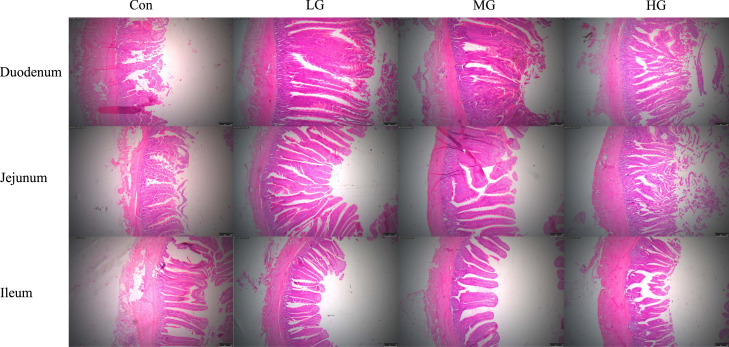
Effects of ZY1 on histopathological changes of duodenum, jejunum, and ileum with H&E staining (original magnification of 40×). Note: Scale bar, 100 μm, *n* = 9.

The results of intestinal morphology are presented in Fig. 3 and Table 5. As illustrated in Fig. 3, the intestinal villi in the Con group exhibited a short, sparse, and generally damaged condition. In contrast, the ZY1 treatment groups showed fewer reduction in the number of breaks, with villi appearing more robust and denser. The intestinal villi were significantly restored, resulting in a healthier intestinal tract compared to that of the Con group. Data presented in Table 5 indicated that supplementation with ZY1 significantly increased both VH and VH/CD ratio of the duodenum compared to the Con group (*P* < 0.05). Notably, the effect was more pronounced in the LG group than in other treatment groups.

**Table 5:** Effects of ZY1 on the intestinal morphological parameters of late laying hens.^1^.

Item	0 (Con)	0.05 % (LG)	0.1 % (MG)	0.2 % (HG)	*P*-value
VH (μm)					
Duodenum	434.073 ± 25.560^c^	739.760 ± 29.430^a^	632.654 ± 24.021^b^	659.912 ± 25.846^b^	<0.001
Jejunum	483.877 ± 51.819	520.459 ± 23.338	533.738 ± 16.081	528.702 ± 21.059	0.673
Ileum	369.545 ± 15.834	396.803 ± 28.654	390.702 ± 30.796	401.783 ± 15.587	0.785
CD (μm)					
Duodenum	101.922 ± 4.956	115.849 ± 7.996	101.016 ± 7.744	114.541 ± 7.650	0.318
Jejunum	91.375 ± 10.828	83.943 ± 7.171	90.821 ± 4.029	93.066 ± 3.314	0.804
Ileum	78.753 ± 6.862	78.296 ± 12.719	69.276 ± 6.227	90.194 ± 3.945	0.357
VH/CD					
Duodenum	4.331 ± 0.284^b^	6.648 ± 0.554^a^	6.678 ± 0.722^a^	5.923 ± 0.375^a^	0.009
Jejunum	5.452 ± 0.422	6.580 ± 0.608	5.953 ± 0.274	5.756 ± 0.345	0.317
Ileum	4.954 ± 0.438	5.668 ± 0.553	5.789 ± 0.380	4.502 ± 0.212	0.113

^a,b^ Within a row, values with no common superscripts indicate a significant difference (*P*<0.05).

Abbreviations: VH, villus height; CD, crypt depth; VH/CD, villus height to crypt depth ratio.

1 Data are expressed as mean ± SEM, *n* = 9.

## Discussion

To date, the role of probiotics as an alternative for antibiotics has been widely implemented in animal feed (Sjofjan et al., 2021; DeLeon et al., 2023; Huang et al., 2024). Previous studies indicated that the addition of *Bacillus subtilis* to feed could enhance poultry production performance, improve immune function, and protect intestinal morphology. However, different strains may produce varying effects (Jha et al., 2020; Reis et al., 2017). This study primarily investigated the effects of *Bacillus subtilis* ZY1 on production performance, egg quality, serum parameters, and intestinal morphology in laying hens.

Our results demonstrated that while ZY1 supplementation did not significantly influence average egg weight, its addition notably increased egg laying rates in both the LG and HG groups, consistent with findings reported by Zhang et al. (2021). This increase may be attributed to enhanced nutrient utilization for egg production due to ZY1 supplementation; however, further research is necessary to elucidate the underlying mechanisms due to lack of supporting data. In this study, the feed-to-egg ratio was significantly reduced in the HG group compared with other groups — a finding corroborated by Zhu et al. (2020). It was speculated that high doses of ZY1 in diets may optimize nutrient utilization to enhance egg production in laying hens (Ray et al., 2022). Additionally, we observed an inverse relationship between qualified egg rate and ZY1 supplementation levels: notably, high-dose groups (HG) did not show a significant reduction in unqualified eggs (sand-shelled, soft-shelled or broken eggs), whereas there was a significant increase observed in both LG and MG groups compared with the Con group. Similar results were also noted by Liu et al. (2024) and Tsai et al. (2023), suggesting that ZY1 could improve vitamin and mineral absorption thereby promoting eggshell formation and enhancing qualification rates for eggs. In summary, incorporating ZY1 into diets for laying hens could significantly improve egg production performance — particularly at lower doses, such as 0.05 %.

Egg quality declines with the advancing age of laying hens, leading to increased breakage during transportation and significant economic losses for farmers. Therefore, enhancing egg quality in laying hens is a crucial strategy for improving farmers' economic benefits (Liu et al., 2023). Previous studies have demonstrated that dietary supplementation with *Bacillus subtilis* can significantly improve eggshell strength, Haugh unit scores, and albumen weight (Yang et al., 2022; Wang et al., 2021; Shi, Zhang and Kim, 2020). In the present study, we observed significant increasing in eggshell weight and yolk weight at the low dose (0.05 % ZY1) and high dose (0.1 % ZY1) of *Bacillus subtilis* ZY1. This suggested that dietary addition of ZY1 could enhance egg quality during laying hens’ production cycle. This improvement may be attributed to enhanced antioxidant properties in laying hens which likely contribute to better eggshell quality through a healthier intestinal barrier and improved albumen quality via reduced lipid and protein oxidation (Chen et al., 2020). Eggshell color serves as one of the most intuitive indicators for consumers assessing egg quality; typically, darker eggshells are more favored by consumers. In this study, dietary supplementation with ZY1 increased eggshell color, which rendered the eggshells dark brown by decreased L* values, increased a* values and b* values (Bi et al., 2018). These results implied that ZY1 could effectively enhance eggshell coloration. Notably, among all treatments tested in this study, the low dose (0.05 % ZY1) of *Bacillus subtilis* ZY1 was found to be most effective regarding egg quality in late-laying hens.

The decrease in resistance to oxidative stress is closely associated with the decline in egg production performance and egg quality during the later stages of laying hens (Zhan et al., 2019). Research has suggested that dietary probiotics can enhance the ability to scavenge free radicals, mitigate damage caused by oxidative stress, and maintain the body's redox balance (Zhao et al., 2023). This study found that laying hens supplemented with ZY1 exhibited significantly increased activities of T-AOC, CAT, GSH-Px and SOD, suggesting an enhanced antioxidant defense system in response to ZY1 supplementation. Concurrently, levels of MDA — a cellular end-product of lipid peroxidation and a critical indicator of oxidative stress — showed a significant reduction (Xiang et al., 2019), further demonstrating the potential of ZY1 to migrate oxidative damage. These results clearly indicated that ZY1 could enhance antioxidant enzyme activity while reducing lipid peroxidation, which may contribute to increased albumen weight and improved overall performance and egg quality in laying hens (Chen et al., 2021). Numerous studies have demonstrated that the addition of *Bacillus subtilis* to poultry feed improves the antioxidant status of laying hens (Zou et al., 2022; Chen et al., 2020), which aligns with our findings.

Immunoglobulins are antibodies which can protect the host from pathogens and other potentially toxic microorganisms, mainly including IgA, IgM, and IgG three classes (Guo et al., 2022). In serum, the enhancement of immune function improves laying hens' ability to adapt to complex environments and maintain their health, thereby enhancing egg quality (Shi et al., 2024). In this experiment, dietary supplementation with ZY1 significantly increased the levels of IgA in both the MG and HG groups compared to the Con group. Furthermore, all three treatment groups exhibited significant increases in serum levels of IgM and IgG relative to the Con group. These findings highlight the potential of ZY1 to strengthen both local and systematical immunity in laying hens during late laying periods. Consistent with numerous studies, these findings demonstrated that dietary supplementation with *Bacillus subtilis* can elevate immunoglobulin levels (Chen et al., 2019, 2023), indicating that *Bacillus subtilis* has a positive effect on enhancing immunity in laying hens. This probably attributed to its antioxidant mechanisms.

Serum biochemical indices are key indicators reflecting the metabolism and health status of poultry (Song et al., 2023). The levels of AST and ALT in serum serve as parameters for assessing liver injury (Sindaye et al., 2023). In our study, dietary supplementation with ZY1 significantly reduced AST levels alongside a notable decrease in ALT content within the HG group, suggesting that ZY1 might enhance the overall health of laying hens. These results are consistent with previous studies indicating that dietary *Bacillus subtilis* is beneficial for improving liver function in poultry (Liu et al., 2022). Furthermore, serum LDH reflects cellular metabolic status, while TG is associated with lipid metabolism (Sun et al., 2020). In this study, ZY1 supplementation resulted in a quadratic reduction in both LDH and TG levels, particularly at lower doses compared to the Con group. This finding suggested that dietary ZY1 may help mitigate hepatic steatosis (Fernandez et al., 1994) and reduce intestinal lipid absorption (Li et al., 2023). The increased calcium content in the serum of laying hens contributes to eggshell formation. However, mineral absorption can be limited by decreased availability during late laying periods due to intestinal constraints (Zhan et al., 2019). Our findings revealed that ZY1 supplementation significantly elevated calcium levels compared to the Con group, consistent with prior reports (Souza et al., 2021). In conclusion, these results indicated that dietary supplementation with ZY1 is beneficial for enhancing egg production and quality. Additionally, this improvement may be attributed to enhanced immunity and antioxidant capacity.

The integrity of the intestinal barrier is directly associated with redox imbalance and the decline in immune function during the late laying period (Yao et al., 2023; Bai et al., 2018). VH, CD, and the VH/CD ratio are critical indices for evaluating intestinal health in animals, as they directly influence digestive and absorptive functions (Guo et al., 2021; Kim et al., 2022). In this study, we observed that dietary supplementation with ZY1 significantly increased both villus height and the VH/CD ratio in the duodenum. These results suggested that ZY1 supplementation may enhance digestive and absorptive functions by increasing the surface area of the intestinal lining and improving mucosal structure (Wang et al., 2022; Abdelqader, Al-Fataftah and Das, 2013). Previous studies have reported similar findings to those presented here (Zou et al., 2022).

## Conclusions

In summary, dietary supplementation with a dosage of 0.05 % *Bacillus subtilis* ZY1 could improve production performance and egg quality, while strengthening antioxidant capacity and immune function, and promoting overall metabolism. Additionally, 0.05 % ZY1 administration could increase VH and VH/CD in the duodenum, leading to improved intestinal health. These findings may facilitate the development of a novel green feed additive and provide insights into the rationalization of ZY1 utilization in late-laying hens.

## Disclosures

The authors have no conflicts of interest to declare regarding this research.

## Declaration of competing interest

The authors declare that they have no known competing financial interests or personal relationships that could have appeared to influence the work reported in this paper.

## Appendix Supplementary materials

Image, application 1
