# Supplementary material for: Effects of Bacillus subtilis ZY1 on production performance, egg quality, serum parameters and intestinal health in laying hens
Source: Poult Sci. 2025 Apr 1;104(7):105120. doi: 10.1016/j.psj.2025.105120 (PMC12099773; doi:10.1016/j.psj.2025.105120)
Supplement: Supplementary file 1 [file mmc1.pdf]

生物医学伦理审查证明

国家自然科学基金委员会：

按照贵委对项目申报有关学术伦理的相关要求，我校组织相关专家对周晓辉同志申请的基金项目且可能涉及生物医学伦理学的相关申报项目进行了伦理审查。

经审查认为，我单位周晓辉同志申报的河北省自然科学基金委生物医药联合基金重点项目《防治畜禽腹泻抗菌蛋白 CpxP 的抑菌活性及其机理》中，有关实验研究涉及实验动物的生物医学伦理学范畴。课题组及负责人承诺在进行该项目研究中严格按照“贵委有关学术伦理”相关规定，遵守有关法律和规章制度，保证相关研究工作符合人类（动物）的道德伦理标准和国际惯例，切实维护被试对象及有关人员的权益，并及时向我校科研管理部门汇报相关进展。学校也将适时监督该项目严格按照有关规范和标准开展实验研究。

特此证明。

河北科技大学学术委员会

河北科技大学(代章)

主任(签字)：胡小琳

2020 年 3 月 13 日
